# Peer effects in health valuation: the relation between rating of contemporaries’ health and own health

**DOI:** 10.1186/s12955-018-0978-8

**Published:** 2018-07-28

**Authors:** Arthur E. Attema, Werner B. F. Brouwer, Jose Luis Pinto Prades

**Affiliations:** 1 Erasmus School of Health Policy & Management (ESHPM), Erasmus University, P.O. Box 1738, 3000 DR Rotterdam, the Netherlands; 2 ESHPM, Erasmus University, Rotterdam, The Netherlands; 3 Department of Economics, Universidad de Navarra, Pamplona, Spain; 4 Yunus Centre for Social Business & Health, Glasgow Caledonian University, Glasgow, UK

**Keywords:** Quality-adjusted life year, Self-rated health, Social comparison, Visual analogue scale

## Abstract

**Background:**

Most health valuation studies assume that individuals’ health valuations do not depend on social comparisons. However, there is some evidence that this assumption is not satisfied in practice. This paper tests whether self-rated health by means of a Visual Analogue Scale (VAS) is related to how one perceives the health of one’s contemporaries, while accounting for one’s health as classified by the EQ-5D classification system.

**Methods:**

In a large sample (*n* = 1500), representative of the general public, we use a VAS to rate respondents’ own health and their assessment of their contemporaries’ health. In addition, we directly ask them whether they perceive their health to be better, the same, or worse than their contemporaries, and we measure their own health according to the EQ-5D-5 L.

**Results:**

We find a positive relationship between own health rating and contemporaries’ health rating, after controlling for the respondents’ own health as classified according to the EQ-5D. Furthermore, we observe a discrepancy between relative health vis-à-vis age peers as measured by an ordinal comparison and relative health as measured by a VAS. Finally, respondents, especially women, tended to overestimate the health of other people of their age.

**Conclusions:**

We provide evidence that people’s own health rating is related to the perception of health of contemporaries. Our results indicate that knowledge about a respondent’s perception of others’ health is useful in explaining health state valuations.

## Introduction

It is increasingly recognized that reference points, a crucial element in prospect theory, play an important role in valuations, including health state valuations. The nature and source of such reference points remain less well understood. One possible natural reference point in evaluating own health, as done in self-assessed health, may be the health of relevant others. Individuals are indeed sometimes asked to (indirectly) indicate if they believe their health to be better or worse than that of same-aged peers, using ordinal classification systems such as Likert scales (very good, good, etc.) [1, 2]. These studies have reported moderate correlations between valuations of own health and their relative position compared to others, suggesting that subjective health assessments may be partly based on social comparison [1, 2].

This paper studies the extent to which own health valuations using a Visual Analogue Scale (VAS) depend on how people perceive their health to be in relation to that of others. The VAS is one of the main tools used in health status measurement, both at the individual and the social level. Although there is some debate about the cardinal/ordinal interpretation of measurements conducted using a VAS scale as well as about its direct role in economic evaluations of health care interventions [3], it is used regularly in surveys aimed at measuring and valuing population health [4–9].

If subjective health assessments are partly based on social comparisons, this may have important implications. It may for instance help to explain the stability of people’s own health ratings when growing older, despite increasing physical problems [10–12]. That is, if elderly people are more likely to compare themselves to other elderly, who on average are in poorer health than younger individuals, their relative position within this comparator group may cause them to rate their health state more favorably than when judged in an absolute way or when compared to younger people.

It may also have important implications about the way that the social value of health treatments is estimated. For example, assume that data from cancer patients have been collected in a randomized controlled trial and their utilities are estimated using the tariff on the EQ-5D. Assume that these patients are asked to place themselves in one of the five categories within the “pain” domain. If their reference point is how they compare themselves against other cancer patients, they may place themselves in level 2 or 3, implying they do not have very important problems of severity. This will reduce the health benefit of reducing pain. This argument is different from the “adaptation” one. If it is the case that subjects adapt, then their utility level is the utility they state. However, in our example, they state that their pain is mild because they are lowering the threshold of “mild pain” given that they compare with the pain of others but they are not feeling less pain, which is the case with people who adapt to their circumstances. In general, if people respond to health questions using reference points, the measurement of health benefits would be more complex than usually thought.

This may also be relevant in the context of economic evaluations. Fryback and Lawrence [13] already suggested that the benefit of medical treatments for old people may be exaggerated when economic evaluations measure the benefit from treatments as if successful treatment returns all people to full health. Many people have other illnesses as well, which is especially true for elderly, making the endpoint of full health unrealistic. Depending on the way health state valuations are derived and used (e.g. VAS or Time Trade-Off, patient or general public valuations, population averages or patient sample specific scores), an influence of social comparisons may impact results of economic evaluations as well. If older patients give higher average values than young people for the same health state, this may potentially lead to larger gains in modelling exercises using such values. The opposite holds for benefits from interventions that improve quality of life to full health without affecting life expectancy. These will be underestimated if elderly people value their impaired health higher. It matters therefore whether respondents value their health in comparison to relevant others. The term “relevant others” intentionally is left vague here since it is not clear who those people can be. Although it would be good to have more information on which people subjects compare themselves with when valuing their health, this paper will focus on investigating the existence of such comparisons with same-aged peers.

Our study is the first to include a (VAS) scale to measure health status in a social comparison context, testing whether these VAS valuations reflect the relative position of own health. We do this in a representative sample of 1500 respondents from the general public, who completed an online questionnaire. Given this large database we are able to perform some further tests based on the literature review reported in section “Background”. We argue in this section that the higher relative rating of health among elderly also holds when we use the VAS. The results are shown in section “Results” and discussed in section “Discussion”. Finally, section “Conclusions” concludes.

## Background

*Reference group theory*, which assumes that subjective assessment of health depends on the individual’s comparison group, is often used to explain elderly persons’ positive health assessments. According to this theory, elderly adults adopt to their situation by maintaining positive health perceptions when confronting illness; they adjust their perceptions of health in relation to peers of their age [14, 15], or to their past health [16, 17]. This paradox is known as the *paradox of aging* or the *well-being paradox* [18].

Social comparison may be a strategy that protects older people from the negative effects of aging. Brandtstädter et al. [19, 20] propose a dual-process framework to describe how aging individuals reduce discrepancies between their current and ideal situations. One process is deemed the *assimilative mode*, comprising “efforts and intentional activities to modify the actual situation to attain a closer fit with personal goals and projects” [19]. The other process is the *accommodative mode*, which “comprises mechanisms and processes by which goals and projects are adjusted to available resources of action” [19]. According to this theory, health deteriorations due to age can be regarded as losses in resources, leading people to favor the accommodative process over the assimilative one as they get older [21].

Empirical evidence supporting this theory was reported by Heckhausen and Krueger [22] and Heckhausen and Brim [23], who found that older persons were more inclined to have a favorable self-view than younger persons. In addition, Baron-Epel and Kaplan [2] asked respondents to describe their health status on a 5-point Likert scale and repeated this question while asking respondents to compare their health to the health of men/women of their age. They found that respondents aged 65–75 reported better health when valuing their own health compared to age peers than when valuing their own health without this comparison, and no differences in these measures for younger age groups.

Cheng et al. [24] studied whether, among a sample of Chinese adults, perceiving one’s own physical health as better than the physical health of contemporaries has a stronger increasing effect on self-reported health (SRH) in older than in younger persons. Moreover, they studied whether such a perception could account for the relative stability of SRH in later life. Both showed a positive impact of social rating, and this impact became stronger the older the respondent. In addition, Cheng et al. [24] observed a tendency to be optimistic about own health compared to health of contemporaries, and this effect also increased with age, even after controlling for physical symptoms and the interaction of physical symptoms and age. Consequently, older persons did not rely as much on the extent of their physical symptoms to assess their SRH as younger persons, which is consistent with an increasing gap between subjective and objective health [25, 26].

Eriksson et al. [27] directly asked respondents to indicate whether they assessed their health to be better, worse or the same as others of their age. It turned out that respondents, especially men, tend to overestimate their health in relation to others when age increases. This implies an ‘over-adjustment’ for age. Eriksson et al. [27] therefore argue that age-comparative measures may be less suitable if one aims to study changes in SRH longitudinally. They conclude that subjective health shows a weak correlation with age, and suggest it is to some extent assessed according to what can be expected given the circumstances.

## Methods

In summary, most of the evidence reviewed suggests that people at least partly base their own health rating on age and on health of same-aged peers. The main objective of this study is to test if VAS valuations reflect the relative position of own health of subjects in society, while correcting for health problems as reported according to EuroQol Classification System (https://euroqol.org/). The hypotheses we want to test in this paper are as follows. First, we hypothesize that the respondents’ own health rating will deteriorate with increasing age at a lower rate than their rating of contemporaries’ health. Similarly, we expect the proportion of respondents who consider themselves to be in a better health state (when asked directly) than their contemporaries to increase with age (Hypothesis 1). As a consistency check, we test if these respondents reporting to be in better health indeed also rate their own health higher than they rate the health of their contemporaries on the VAS. Second, we hypothesize that respondents, when reporting their own health state, incorporate concerns about others and coping effects, and, hence, that their VAS will be related to how they rate the health of contemporaries (Hypothesis 2).

In this paper we combine the data of three separate online surveys, administered by the authors of this paper in 2013 and 2014. Two of these are reported elsewhere [28, 29]. The three surveys aimed to elicit individual and societal preferences for health outcomes^1^ and all contained a number of questions about relative health (see Table 5), which were not used for the aforementioned papers. These questions were posed at the end of each survey.^2^ As a result, we obtained a large dataset of 1500 respondents. All three surveys were representative of the Dutch adult population in many characteristics. These respondents were randomly drawn from an internet panel, recruited by Survey Sampling International. Table 1 summarizes some demographic data. In all three surveys, the VAS tasks were asked at the start.

**Table 1:** Demographic characteristics of the study sample*

Characteristic	Category	Count	Percentage
Age (mean: 45.1, s.d. 15.0)	18–30	330	22
	31–40	219	14.6
	41–59	333	22.2
	51–60	359	23.93
	61–75	259	17.27
Sex	Male	736	49.1
	Female	764	50.9
Monthly income before tax	0-€1999	591	39.4
	€2000–€2999	385	25.67
	€3000–€3999	277	18.47
	€4000 and more	247	16.47
Education	Low (primary school/secondary school, lower level)	386	25.73
	Medium (Secondary school, higher level/secondary vocational education	608	40.53
	High (higher professional education/university)	506	33.73
Marital status	Single	359	23.93
	Married	682	45.47
	Living together, not married	219	14.6
	Divorced	161	10.73
	Widowed or other	79	5.27

*The results do not always add up to 100% because of rounding

For this study, we gathered the following data^3^: subjective feeling of own health today as measured by a visual analogue scale (VAS_OWN), own health status in terms of the EQ-5D-5 L, the respondents’ estimation of their contemporaries’ health status (i.e., other people of their age) as measured by a VAS (VAS_OTHER), and a related ordinal question asking whether the respondent felt their health was better than, worse than or the same as their contemporaries’ health (SOC_COMP).

The EQ-5D-5 L describes health according to five dimensions, each consisting of five levels [30]. We used the EQ-5D because it is a standardized instrument for measuring generic health status, which is currently one of the leading instruments to determine national health state tariffs in many countries [31–34]. Because the EQ-5D gives a discrete measure of the health status, we first have to transform this measure in order to make it analytically tractable. We chose to use the Misery Index (MIS_INDEX) for this, which is often used and equals the sum of the five digits describing each health state, with a higher number representing a worse health state [35–37]. For example, the misery index of EQ-5D state 23245 would equal 16.

These variables allow us to test the hypotheses formulated in Section “Background” as follows. Hypothesis 1 is tested by estimating the correlation between age and the difference between VAS_OWN and VAS_OTHER. In addition, we perform a logistic regression of a transformation of SOC_COMP on age, where we transform SOC_COMP into a dummy variable BETTER_HEALTH, which equals 1 (0) if the respondent indicated to be in better (same or worse) health than contemporaries.

We perform the consistency check by splitting the sample into three groups depending on the respondent’s answer to SOC_COMP and computing the difference between VAS_OWN and VAS_OTHER. Then, for each of these groups, we test whether the sign of this difference corresponds to their answer to SOC_COMP. According to our hypothesis, respondents who indicate to be in better [worse] health than their age peers in SOC_COMP, will rate VAS_OWN significantly higher [lower] than VAS_OTHER.

The test of Hypothesis 2 involves a regression of VAS_OWN on VAS_OTHER and several other demographic variables, including MIS_INDEX, age, gender, income, and education, in order to control for possible confounding effects that influence both VAS_OWN and VAS_OTHER. For example, it may be that variables such as MIS_INDEX influence both VAS_OWN and VAS_OTHER, in which case omitting this variable may result in a spurious effect of VAS_OTHER on VAS_OWN.

## Results

As can be seen in Table 2, VAS_OWN is considerably lower than VAS_OTHER (paired t-test, *p* < 0.01). One contribution of this paper is to split this effect into two components. On the one hand, more people think that their health is worse than that of their contemporaries than the opposite. Those people tend to give higher values in the VAS to others than to themselves, as it could be expected. On the other hand, those who state that their health is better than their contemporaries, do not translate this into different values in the VAS.

**Table 2:** Average health assessments decomposed into ordinal relative health position (SOC_COMP)

	Full Sample			
	SOC_COMP			
	Better	Same	Worse	All
VAS_OW (S.E.)	0.85 (0.01)	0.83 (0.00)	0.65 (0.01)	0.78 (0.00)
VAS_OTHER (S.E.)	0.86 (0.01)	0.86^a^ (0.00)	0.85^a^ (0.01)	0.86^a^ (0.00)
*p*-value	0.64	< 0.01	< 0.01	< 0.01
N	352	656	492	1500
Percentage	23.5%	43.7%	32.8%	100%

^a^Different from VAS_OWN at the 1%-significance level (paired t-test)

We find a negative correlation between age and VAS_OWN, confirming the common finding that health feelings deteriorate with age, although the size of the correlation coefficient is low (Kendall’s τ = − 0.055, *p* < 0.01). The logistic regression of the binary variable BETTER_HEALTH (‘being in better health than contemporaries or not’) revealed a positive relation with age, in line with Hypothesis 1, but this result was not significant (only at a 10%-significance level with *p* = 0.09). Table 3 decomposes the results according to aforementioned three age classes. This makes clear that the gap between own health and that of others (with own health lower than others’ health) was largest in middle-aged respondents compared to younger and older respondents. Hence, according to this test, Hypothesis 1 is partly rejected; instead of a negative relation between age and VAS_OTHER-VAS_OWN, we observe an inverse U-shaped pattern. However, the correlation coefficient between age and the gap between VAS_OTHER and VAS_OWN is significantly negative (Kendall’s τ = − 0.051, *p* < 0.01), which supports Hypothesis 1. In addition, we performed a regression of this gap on age and age squared, which showed a positive coefficient for age (1.05, *p* < 0.01) and negative coefficient for age squared (− 0.01, *p* < 0.01), confirming the inverse-U relation.

**Table 3:** Average health assessments decomposed into age classes and ordinal relative health position (SOC_COMP)

	Young (18–35)				Middle (36–55)				Old (56–75)			
	Better	Same	Worse	All	Better	Same	Worse	All	Better	Same	Worse	All
A: VAS_OWN (S.E.)	0.88 (0.01)	0.84 (0.01)	0.71 (0.02)	0.81 (0.15)	0.84 (0.01)	0.82 (0.01)	0.63 (0.01)	0.75 (0.18)	0.85 (0.01)	0.82 (0.01)	0.65 (0.01)	0.78 (0.16)
B: VAS_OTHER (S.E.)	0.88 (0.02)	0.88 (0.01)	0.89 (0.01)	0.88 (0.14)	0.86 (0.01)	0.87 (0.01)	0.86 (0.01)	0.86 (0.11)	0.84 (0.01)	0.84 (0.01)	0.81 (0.01)	0.83 (0.11)
A – B (95% C.I.)	0.01 (−0.03–0.04)	−0.04 (− 0.06 – − 0.01)	−0.18 (− 0.21 – − 0.15)	−0.07 (− 0.09 – − 0.52)	−0.02 (− 0.04–0.00)	−0.05 (− 0.06 – − 0.03)	−0.23 (− 0.26 – − 0.20)	−0.11 (− 0.12 – − 0.09)	0.01 (− 0.02–0.03)	−0.02 (− 0.03–0.00)	−0.16 (− 0.19 – − 0.13)	−0.05 (− 0.07 – − 0.04)
N	97	202	126	425	135	272	239	646	120	182	127	429
Percentage	23%	48%	30%	100%	21%	42%	37%	100%	28%	42%	30%	100%

There was no difference between VAS_OWN and VAS_OTHER for those respondents indicating their health to be better than their contemporaries in SOC_COMP (Table 2, averages: 0.85 vs. 0.86; paired t-test: *p* = 0.63). VAS_OWN was lower than VAS_OTHER for both respondents perceiving their health to be the same as their contemporaries (averages: 0.83 vs. 0.86; paired t-test: *p* < 0.01), and respondents perceiving it to be worse (averages: 0.65 vs. 0.85; paired t-test: *p* < 0.01). As a result, the consistency check reveals that VAS values are consistent with SOC_COMP for respondents indicating their health to be worse than contemporaries in SOC_COMP, but not for those indicating it to be the same or better. This seems to be caused by a general tendency to be pessimistic about one’s own health compared to peers’ health in the VAS tasks. When splitting the dataset into three age groups (18–35, 36–55, 56–75), we found VAS_OWN to be lower than VAS_OTHER for each separate age class as well (paired t-tests: all p < 0.01), making this finding universal across age.

Because the residuals were heteroscedastic (White’s test, *p* < 0.01), we conducted a regression with White heteroscedasticity-consistent standard errors to test Hypothesis 2. Table 4 shows the results, after removing all insignificant variables. VAS_OTHER has a positive sign and is highly significant; hence, Hypothesis 2 is confirmed, indicating that relative positions matter for VAS ratings. The correlation of this variable is robust to the inclusion of MIS_INDEX, which as expected also has a strong negative association with VAS_OWN. The results of a Tobin’s censored regression, allowing for the non-normal distribution of VAS_OWN (Kolmogorov-Smirnov test, p < 0.01) are reported in the Appendix, which, reassuringly, are similar.

**Table 4:** White heteroscedasticity-consistent standard errors least squares regression, dependent variable: Own health rating (VAS_OWN, *n* = 1500)

	Coefficient	*p*-value
Constant	85.34	< 0.01
VAS_OTHER	0.23	< 0.01
MIS_INDEX	−3.84	< 0.01

## Discussion

In this paper we have studied the relation between age and health rating, as well as the effect of one’s perception of the health state of contemporaries on one’s own health rating for different ages. First, we observed a significant, but weak, negative correlation between age and own health rating, confirming previous findings [27]. Second, elderly were somewhat more likely than the young to classify their health to be better than their contemporaries’ health. This implies that elderly are less pessimistic with respect to their own health vis-à-vis contemporaries than younger respondents. Third, we found a discrepancy between relative health as measured by an ordinal comparison and relative health as measured by a VAS. That is, people who indicated their health to be the same as [better than] their contemporaries’ health in the ordinal comparison, on average rated their own health lower than [the same as] the health of their contemporaries in a VAS. Hence, there appears to be an inherent bias in the VAS in reflecting the ordering of one’s health and their contemporaries’ health, which may be due to either the VAS of own health being an underestimation, the VAS of contemporaries’ health being an overestimation, or some combination thereof. It could therefore be that the VAS does not accurately reflect perceptions of quality of life.

A further explanation may be based on the different health distributions in same-sex age peers for men and for women. Because relatively many older men are in good health, as long as they are alive, men are more likely to compare themselves with healthy peers of the same age. In contrast, relatively many older women have health problems. Therefore, a woman comparing her health to that of her peers of the same age is more likely to make the comparison with other women with health problems, other things being equal [1, 38]. Indeed, women have been shown to be more likely to compare themselves to peers with health problems than men are [38]. It is also important to emphasize that our sample also consisted of adults younger than 55 years, which in general would be expected to be fairly healthy. The fact that our sample contained relatively few older people also implies that it is unlikely that our results were influenced by the ‘absence of contemporaries’ argument.

From previous research on social comparison in health, it was already known that people tend to lower their peers’ health condition relative to their own as they get older, causing them to stand out more favorably [24]. Related to this, there is ample evidence that older individuals’ own health rating decreases much less steeply with age than would be expected from their growing number of health problems [11, 12]. Our study confirmed these behavioral traits, in that own VAS rating decreased considerably less quickly with age than the VAS rating of contemporaries, but also added to this evidence base in a number of ways, as described before. However, in contrast to earlier studies, respondents in our study tended to overestimate the health of other people of their age. In addition, the rating of their own health state was positively related to their rating of their contemporaries’ health, even after controlling for the respondents’ own health as classified according to the EQ-5D. An alternative explanation for the positive correlation between own VAS rating and VAS rating of contemporaries would be that people rate health high or low in general. However, taking into account the other results indicating that health valuations are reference dependent, we think the interpretation we provide is highly plausible.

It needs noting that the presented study has some limitations. First, given that we used an internet survey, the number of respondents older than 65 years was restricted. Second, only a rating scale was used in our comparison. Future research is called for to replicate this study using choice-based valuation methods such as Time Trade-Off and Standard Gamble. Third, respondents always valued their own health before valuing the health of their age peers. Finally, respondents might have had difficulty in determining whom to take into account when rating the health of other people in their age group, which may have hampered comparability.

Notwithstanding these limitations, some important implications of our findings need to be stressed. First, an implication of the relation between health ratings of contemporaries and of oneself is that the same EQ-5D profiles in health state measurements may be valued differently by different individuals, depending on their estimations of their age peers’ health. Hence, one has to make an attempt to correct for this influence, for instance by adding questions about health of peers to an individual health assessment. Second, our results are relevant for economic evaluations. For instance, the observed effect of social comparisons may cause too high expected QALY gains from life-extending treatments of elderly patients; on the other hand, interventions that bring back quality of life to full health without increasing life expectancy will appear less cost-effective in older patients. This means that health policy makers may wish to give more weight on quality-of-life-enhancing treatments relative to life-extending treatments. Finally, our results suggest that one reference point of respondents is the health of others similar to themselves, making health benefit measurements more complex. This also implies that public health research needs to take into account these negative externalities caused by an improved average health status. There may of course also be other relevant reference points, which could be explored in future research.

## Conclusions

We believe our study has provided convincing evidence that people’s self-assessed health is related to the perception of health of contemporaries. We encourage further research to investigate whether and how social comparisons affect health state valuations using e.g. Time Trade-Offs or Discrete Choice Experiments. This would also shed more light on the question how these comparisons could affect more common national tariffs (such as for the EQ-5D). In addition, it could be interesting to further investigate whether such comparisons also affect the interpretation of descriptive systems such as that of the EQ-5D, for instance in the definition of mobility and usual activities.

**Table 5:** Description of the constructed variables

Variable	Description	Response levels
VAS_OWN	Subjective feeling of own health today as measured by a visual analogue scale.	Number between 0 and 100, divided by 100 in the analysis to arrive at values between 0 and 1.
VAS_OTHER	The respondents’ estimation of their contemporaries’ health status (i.e., other people of their age) as measured by a VAS.	Number between 0 and 100, divided by 100 in the analysis to arrive at values between 0 and 1.
SOC_COMP	Ordinal question asking whether the respondent felt their health was better than, worse than or the same as their contemporaries’ health.	Better/Worse/Same
BETTER_HEALTH	Dummy variable indicating whether respondent felt their health was better than their contemporaries’ health or not.	1 (better health) or 0 (the same or worse health)
MIS_INDEX	Summation of the levels of each dimension, with a higher number representing a worse health state.	Number between 5 and 25

## Appendix

**Table 6:** Tobit regression, dependent variable: VAS_OWN (*n* = 1500)

	Coefficient	*p*-value
Constant	81.56	< 0.01
VAS_OTHER	0.25	< 0.01
MIS_INDEX	−3.17	< 0.01
SOC_COMP: Own health better than contemporaries’ health	2.18	< 0.01
SOC_COMP: Own health worse than contemporaries’ health	−8.53	< 0.01

## Abbreviations

MIS_INDEX: Misery index
SOC_COMP: Social comparison
SRH: Self-reported health
VAS: Visual analogue scale

## Footnotes

1 One paper [28] is about eliciting preferences for distributions of quality of life among groups (*n* = 517). The second paper [29] is about measuring individual preferences for quality of life in a prospect theory framework (*n* = 463). The third survey (*n* = 520) was a pilot study about preferences for different age groups. This study has not been published and the data have not been reported anywhere.

2 The average duration of each survey was about 20 min.

3 Table 5 gives an overview of the characteristics and response levels of the constructed variables.
